# Evaluation of salivary vasopressin as an acute stress biomarker in healthy dogs with stress due to noise and environmental challenges

**DOI:** 10.1186/s12917-020-02555-5

**Published:** 2020-09-11

**Authors:** Yi-Kyeong Jeong, Ye-In Oh, Kun-Ho Song, Kyoung Won Seo

**Affiliations:** College of Veterinary Medicine, Chungnam National University, 99 Daehak-ro, Yuseong-gu, Daejeon, 34134 Republic of Korea

**Keywords:** Stress measurement, Stress behaviour, Hypothalamus-pituitary-adrenal axis, Arginine vasopressin, Cortisol, Canine, Saliva

## Abstract

**Background:**

Stress is associated with various detrimental changes in physiological health that affect an animal’s quality of life. The hypothalamus-pituitary-adrenal (HPA) axis and the sympathetic-adreno-medullar (SAM) axis are two main physiological pathways that constitute the stress response of an organism. Arginine vasopressin (AVP) is a mediator of the HPA axis and is known to be related to social behaviours and stress. The serum concentration of AVP is higher in more aggressive dogs and humans with post-traumatic stress disorder. Salivary biomarker analysis is a non-invasive method to assess stress. The purpose of this study was to evaluate the possibility of using salivary AVP as an acute stress biomarker in dogs. Salivary AVP concentration was measured before and after exposure to all relevant environmental stimuli (i.e. car trip to the lab, physical examination by the veterinarian, and sampling procedure,) and then after 30 min of vacuum noise exposure. Behavioural assessments, physiologic parameter assessments, and serum cortisol analysis were conducted in combination. Statistical analysis was conducted separately in the total study population, the less stressed group, and the more stressed group, respectively.

**Results:**

Based on stress behaviour analysis scores, 28 dogs were classified into less or more stressed groups. All four physiologic parameters (blood pressure, body temperature, heart rate, and respiratory rate) were significantly increased after noise and environmental challenges, in the more stressed group. Serum cortisol did not show any significant change. Salivary AVP significantly decreased after noise and environmental stimulation in the more stressed group but not in the less stressed group. Salivary AVP and blood pressure changes were negatively correlated in the more stressed group.

**Conclusion:**

Salivary AVP may be a potential acute stress biomarker in dogs.

## Background

Stress refers to a response by an organism to a threat where it concentrates its effort to try to restore the optimal state [1, 2]. Both physiologic and psychologic alterations can be stressors. The stress response of an organism is constituted mainly by two physiological pathways: the hypothalamus-pituitary-adrenal (HPA) axis and the sympathetic-adreno-medullar (SAM) axis. The SAM axis releases epinephrine and norepinephrine, and the HPA axis releases glucocorticoids in response to stress. These mediators alter systemic metabolism and organ function to provide a quick response and restore the organism to its optimal state [3, 4].

Stress influences multiple parts of the body, such as the immune, gastrointestinal, and urogenital systems. It is associated with various injurious changes to physical health, which affect quality of life [5]. There is an abundance of evidence from pre-clinical, clinical, and epidemiological trials substantiating that stress can induce negative consequences in both humans and non-human animals, contributing to increased patient morbidity or mortality [6]. In domesticated dogs, this extends to a shortened lifespan related to anxiety-induced stress, gastric ulceration in racing dogs (as a consequence of exercise stress), and emotionally unstable events resulting in psychodermatoses [7–9]. Thus, measuring and managing stress has become an important issue in both human and veterinary medicine.

As of now, science lacks research methods that can directly quantify individual stress levels [10]. Various indirect methods of measuring stress in humans include self-assessment, several biomarkers, and neuroendocrinology [11, 12]. As in human medicine, diverse methods are attempted to measure the stress of animals in veterinary medicine. Since verbal self-expression of stress is impossible, indirect methods like vital sign changes, behavioural signals, and biomarkers are required [13–15]. Previous studies have extensively described SAM and HPA secretion as stress biomarker candidates. However, there are still limitations such as, instability, diurnal fluctuations, and the intricate relationship between stimulation and secretion, which warrant further investigation of this field.

Cortisol is the final mediator of HPA axis activation and is associated with the mobilisation of energy reserves, stimulation of gluconeogenesis, protein and fat metabolism, and the suppression of temporary undesirable immune responses [16]. Cortisol is stable in various matrices (e.g. blood, saliva, urine, and hair) [11]. Although cortisol concentrations in faeces or hair reflect chronic stress, concentrations in blood or saliva reflect the current circumstances of an organism [17]. Due to this property, cortisol is the most broadly studied biomarker for both acute and chronic stress in veterinary medicine [18–20].

Another mediator of the HPA axis, arginine vasopressin (AVP), is also actively studied as a stress biomarker. AVP is a neuropeptide hormone which is synthesised in the hypothalamus and secreted through the posterior lobe of the pituitary gland. It is the primary activator of the HPA axis and has antidiuretic and vasoconstrictive action [21, 22]. Furthermore, the behavioural role of AVP as a neural regulator of various social behaviours (e.g. aggression, affiliation, pair bonding, etc.) has been described [23]. According to one human study, elevated levels of plasma AVP in patients with post-traumatic stress disorder compared to both traumatised and healthy non-traumatised controls were reported [24]. Similar results exist in veterinary research. Serum AVP levels are elevated after fear provocation [25], aggressive dogs have higher total serum AVP levels than friendly dogs [22], and plasma AVP decreases after affiliative human-animal interactions [26].

Saliva analysis has been widely used in recent stress studies because it is less invasive than blood collection and causes less stress in patients [19, 27]. Similarly, various salivary biomarkers, such as cortisol and salivary alpha amylase (sAA), have been investigated in veterinary medicine [19, 28]. Specifically, methods for the quantification of AVP in saliva have been validated in domestic dogs. Non-extracted salivary samples yielded excellent linearity and parallelism using a commercially available ELISA kit [29].

There are few publications regarding salivary AVP as an acute stress biomarker in dogs. In the latest study of salivary AVP in dogs, dogs diagnosed with separation-related problems showed higher salivary AVP levels than a control group when separated from their owner. This result supports further exploration of the use of salivary AVP as an early, non-invasive biomarker of canine anxiety-related disorders [30].

Salivary AVP has the potential to be an acute stress biomarker in several aspects. First, AVP is elevated along with HPA activation after stress, and its peak time in blood is 15 min, which is faster than that of cortisol [22]. Second, salivary AVP concentrations do not show diurnal fluctuations, unlike salivary cortisol which is known to fluctuate throughout the day and could actually be a confounding factor for biomarker analysis [31, 32].

The purpose of this study was to evaluate salivary AVP as an acute stress biomarker in dogs. We hypothesised that salivary AVP and vital parameters will be increased after a short period of noise exposure and environmental stress challenges. In addition, it was expected that the degree to which AVP and vital parameters were elevated would be in proportion to individual stress level, which would be determined by behavioural analysis.

## Results

### Individual stress level assessment with behavioural analysis

Inter-rater reliability upon reviewing videos was excellent (91.6%).

Oral behaviour was the most frequently presented stress behaviour (100% of dogs), followed by paw lifting (57.1%), vocalisation (53.5%), yawning/panting (50%), and shivering (46.4%), respectively. Each stress behaviour showed great divergence across our sample of dogs. For example, one dog vocalised (barking and whining) 1516 times but did not yawn or shiver at all, while another dog shivered 99.6% of the time (29 min 55 s) but did not vocalise or lift its paws. The number of frequency-counting behaviours was too different, and just summing up each number could not accurately reflect the stress level of dogs. Therefore, each behaviour was scored separately by raw data processing, and the scores were summed to a total score. Based on the total score, dogs with an above-average total score were classified as more stressed and the rest as less stressed group. Fourteen dogs were included in each group.

### Vital sign changes after stress

Physiologic parameters, such as body temperature (BT), heart rate (HR), respiratory rate (RR), and blood pressure (BP), were measured before and after stress induction. If either the pre- or post-RR was panting, it was censored from statistical analysis due to the impossibility of quantification as an exact number. Respiratory data from 5 dogs were censored from statistical analysis, and all 5 dogs were in the more stressed group. Three dogs panted both pre- and post- stress, and two did at post-stress only. Statistical analysis was conducted separately for the total study population, the less stressed group, and the more stressed group. A paired T-test or Wilcoxon signed-rank test was applied according to the normality of the data.

1. Total study population (*n* = 28)

BP (*p* < 0.001) and RR (*p* < 0.001) were significantly increased after stress exposure (Table 1).

2. Less stressed group (*n* = 14)

**Table 1:** Vital changes in total study population

Variable	Pre-stress		Post-stress		*P* value
	Mean ± SD	Median (range)	Mean ± SD	Median (range)	
Blood pressure (mmHg) *	143.2 ± 16.9	138 (116–174)	159.2 ± 28.2	165 (115–197)	< 0.001
Rectal temperature (°C)	38.7 ± 0.42	38.8 (37.9–39.5)	38.7 ± 0.38	38.8 (37.9–39.4)	0.322
Pulse rate (beats/min)	122.5 ± 26.6	128 (80–184)	131.1 ± 29.1	132 (88–188)	0.065
Respiratory rate (breaths/min) * ^a^	26.2 ± 5.3	24 (16–36)	41.5 ± 13.5	40 (20 h)-64)	< 0.001

* Statistically significant (*p* < 0.05) ^a^ panting record censored

Only BP (*p* = 0.003) was significantly increased after stress exposure (Table 2).

3. More stressed group (*n* = 14)

**Table 2:** Vital changes in less stressed group

Variable	Pre-stress		Post-stress		*P* value
	Mean ± SD	Median (range)	Mean ± SD	Median (range)	
Blood pressure (mmHg) *	140.7 ± 16.8	138 (116–174)	157.3 ± 24.7	155 (119–192)	0.003
Rectal temperature (°C)	38.7 ± 0.36	38.8 (38.0–39.2)	38.6 ± 0.35	38.7 (37.9–39.2)	0.690
Pulse rate (beats/min)	120.9 ± 28.0	120 (80–184)	124.3 ± 29.6	120 (88–188)	0.972
Respiratory rate (breaths/min) ^a^	27.0 ± 6.3	24 (16–36)	35.4 ± 12.7	32 (20–64)	0.068

* Statistically significant (*p* < 0.05)

Significant (*p* < 0.05) elevation was observed in all 4 vital parameters (*p* = 0.001 for BP, 0.045 for BT, 0.014 for HR, 0.011 for RR, respectively) (Table 3).

**Table 3:** Vital changes in more stressed group

Variable	Pre-stress		Post-stress		*P* value
	Mean ± SD	Median (range)	Mean ± SD	Median (range)	
Blood pressure (mmHg) *	141.3 ± 14.8	140 (121–162)	153.1 ± 26.3	151 (115–197)	0.001
Rectal temperature (°C) *	38.7 ± 0.53	38.8 (37.9–39.5)	38.9 ± 0.36	39.0 (38.3–39.4)	0.045
Pulse rate (beats/min) *	135.7 ± 22.7	138 (100–168)	142.25 ± 26.4	140 (114–180)	0.014
Respiratory rate (breaths/min) * ^a^	25.0 ± 2.8	24 (20–28)	51.5 ± 8.1	54 (40–60)	0.011

* Statistically significant (*p* < 0.05) ^a^ panting record censored

### Salivary AVP concentration

A Wilcoxon signed-rank test was applied for comparison of two timepoints. Salivary AVP concentration decreased after stress in 21 of 28 dogs in the total study population (66.6%), 9 of 14 in the less stressed group (64.3%), and 12 of 14 in the more stressed group (85.7%).

In the more stressed group, a significant difference was seen between pre- and post-stress salivary AVP concentrations, but not in the less stressed group (Table 4).

**Table 4:** Pre and post stress salivary vasopressin concentration (pg/mL)

Variable	Pre-stress	Post-stress	*P* value
	Mean ± SD	Mean ± SD	
Total study population (*n* = 28) *	80.35 ± 55.46	65.00 ± 43.14	0.003
Less stressed group (*n* = 14)	82.61 ± 68.77	65.81 ± 53.50	0.068
More stressed group (*n* = 14) *	78.07 ± 25.87	63.69 ± 20.16	0.022

* Statistically significant (*p* < 0.05)

### Serum cortisol level

A Wilcoxon signed-rank test was applied for comparison of two timepoints. In both groups, there were no significant differences between pre- and post-stress serum cortisol levels (Table 5).

**Table 5:** Pre and post stress serum cortisol concentration (μg/dL)

Variable	Pre-stress	Post-stress	*P* value
	Mean ± SD	Mean ± SD	
Total study population (*n* = 28)	3.00 ± 3.05	3.37 ± 4.08	0.603
Less stressed group (*n* = 14)	2.62 ± 0.59	3.08 ± 0.67	0.463
More stressed group (*n* = 14)	7.41 ± 3.58	4.89 ± 5.61	0.972

### Statistical correlation between parameters

There was a significant negative correlation between salivary AVP concentration and BP in the more stressed group (R = -0.550, *p* = 0.042). No other parameters were significantly correlated in either group.

## Discussion

The goal of this study was to assess the potential of salivary AVP as an acute stress biomarker in dogs. Current studies of stress in dogs are more focused on the effect of chronic stress, and the effect of acute stress on health is relatively less studied. In acute psychogenic stress situations, cardiac T wave alternans is observed in normal dogs, which implies a potential role of acute stress as a risk factor for cardiovascular malfunction [33]. In human medicine, detrimental effects of acute stress include wound healing delay, atrial fibrillation triggered by T wave alternans, hypercoagulability, and thrombotic tendencies are documented [34–36]. As a part of a natural self-protection mechanism, acute stress has an immunoenhancing role, but when acute stressors become chronic, the effect of stress on the immune system may shift from immunoenhancement to immunosuppression [4, 37]. Therefore, the early detection and amelioration of stress is important not only for quality of life but also for health.

To date, there is no established method to detect acute stress in dogs. Catecholamine secretion after SAM axis activation is very rapid (1 min) and lasts only a few minutes [38]. The brief duration and unstable nature of catecholamines renders them difficult to use as a biomarker in clinical practice. Recently, salivary chromogranin A (CgA), which is co-released with catecholamines, has been investigated as a possible acute stress biomarker in dogs. After a modified Ainsworth’s strange situation test, salivary CgA is decreased. This was interpreted to mean the process of coming to a novel environment may have actually been more “stressful” or “arousing” than the test itself [39]. The sAA is another representative of SAM activation, and has been investigated in dogs. The activity of sAA increased in dogs with various diseases compared to healthy dogs [28].

Salivary immunoglobulin A (sIgA) is a proven stress biomarker in human medicine [40]. It is known to be negatively correlated with cortisol concentration after acute stress in adult dogs but not in puppies [41]. In addition, sIgA has significant diurnal variation, which renders it less useful as a biomarker.

Stress initiates a cascade of hormonal signalling including prompting the hypothalamus to release two important hormones: corticotropin-releasing factor (CRF) and AVP. When CRF and AVP bind to their respective receptors in the anterior pituitary gland, adrenocorticotropic hormone is released, stimulating the release of cortisol from the adrenal glands [42]. Inevitably, AVP is released prior to cortisol and has a shorter peak time (15 min).

We hypothesised that salivary AVP would increase after an acute stress exposure, as it does in blood, but the result of this study was contrary to our hypothesis. Salivary AVP concentration was significantly decreased after stress in the more stressed group. According to one study [22], dogs with a history of aggression had lower free AVP but higher total plasma AVP than matched controls. Furthermore, a recent human study done in patients with perioperative anxiety, demonstrated that there is a weak correlation between AVP concentrations in saliva, blood, and cerebrospinal fluid (CSF), which casts some doubt on the role of salivary, or blood, AVP levels as a representation of central AVP activity [43].

Considering the process of saliva formation, small lipid-soluble molecules, like steroids, can pass relatively easily through cells in salivary glands and diffuse into the saliva from circulating blood. The correlation between saliva and blood cortisol levels has been presented in several studies [19, 44, 45]. However, AVP is a linear peptide and the correlation between saliva and blood concentrations has yet to be elucidated. It is also unknown whether there is a difference between total and free AVP concentrations in saliva under stressful conditions.

Another implication about the result is AVP also acts as an anti-diuretic hormone which is involved BP regulation. A recently published paper stated that the AVP - atrial natriuretic peptide (ANP) - renin-angiotensin-aldosterone-system loops control blood pressure precisely. When AVP activity increases BP enough, atrial stretch receptors stimulate ANP release which in turn, inhibits further AVP release [46]. The exact timing of this regulation, and how it reflects salivary AVP concentration, is still unknown. However, if stress-induced BP elevation is enough to stimulate negative feedback of AVP secretion, this could be a possible explanation for the result.

As a final mediator of HPA axis activation, cortisol rises to its peak level 30–40 min after exposure to stressors [4]. Our study stressors did not elicit significant increases in serum cortisol. This may reflect the relatively slow reactivity of the HPA axis. The intricate mechanism of HPA axis activation can be another interpretation of the results. Activated cortisol secretion may reflect not only stress but physical or psychological arousal or positive excitement [47, 48]. It is also known that different stressors elicit various levels of endocrine activation in humans and in rodent models [49, 50]. Though noise stimulation was established as the major stressor in the present study, there was a possibility of other stimuli including strange environments, transportation, and sampling procedures, which may affect each dog’s stress level separately (e.g. confronting a strange researcher may be more stressful than 30 min of vacuum noise).

In addition, some experimental limitations may have affected the results. Canine salivary cortisol represents the free non-protein-bound fraction in plasma which is biologically active [45]. Thus, measuring cortisol concentrations in saliva is considered a better method to assess adrenocortical function than in serum [19]. However, since the majority of dogs in our study population were small breeds, the saliva collected was not enough for multiple biomarker analysis. As such, serum cortisol was measured instead of salivary cortisol in this study.

Furthermore, 14 dogs (6 in the less and 8 in the more stressed group) had high baseline cortisol concentrations compare to the laboratory reference (< 1.8 μg/dL). Although the dog owners were guided to avoid stressful situations for dogs at home before the experiment, this could not be completely controlled by researchers. Lastly, despite the exclusion of any clinically ill dogs using a prior online survey and routine physical and blood examination, there was a small possibility that dogs with early hyperadrenocorticism without clinical signs could have been included.

Stress is complex and its measurement by a single method is difficult. It is recommended to utilise multiple parameters simultaneously to improve the quality of measurement [17, 51]. Behavioural analysis is widely utilised to assess stress levels of animals. Common stress behaviours of dogs suggested in several other studies are vocalisation, oral behaviour, paw lifting, body shaking, and panting, among others [52, 53]. One study reported that a stress behaviour visual analog scale was useful to reflect stress levels [13], while another suggested the simple summing up of the number of each stress behaviour did not show a correlation with stress biomarkers [53]. In this study, dogs exhibited various degrees of stress behaviours. It is unknown whether one behaviour is superior for reflecting stress than another and there is no standardised technique for behaviour analysis in dogs. The authors agonised over this subject but concluded that one behaviour cannot be weighted more over another because each dog has a different personality, experience, and way of expression. Thus, each defined stress behaviour in this study was assumed to reflect stress equally. This equality assumption of behaviours does not mean numerical equality. We assumed it to be inter-behavioural equality with each behaviour having a different numerical range. Therefore, we graded each behaviour separately to make each behaviour weight equal. For example, as mentioned in the results, a dog vocalised 1516 times but did not yawn or shiver at all while another shivered 99.6% of time but did not vocalise at all. If a simple summing-up of behaviours were used, each dogs’ stress scores would be 1516 and 99.6, respectively, but it was not known if vocalisation is a superior reflection of stress than shivering. After grading each behaviour separately by the scoring process described in the methods, the dogs received 11 and 10 points respectively and both were classified into the more stressed group.

All dogs were classified by this data processing method. The classification into two stress groups (more and less stressed groups) by this method was nearly consistent with the researcher’s subjective impression of each dog’s stress level during a stress session. However, this scoring process has certain drawbacks. This scoring system was developed specifically for this study and its utility has not been evaluated. The grading range of each behaviour was drawn from a small sample size. This method should be evaluated through further research.

According to a study on physiological change and stress, healthy dogs present significantly higher HR, BP, BT, and panting tendency in a veterinary hospital compared to their home environment [54]. These physiologic changes were interpreted to be a result of stress from clinical processes and to be similar to the “white coat effect” in humans. In the more stressed group, increases in vital parameters were more obvious than in the less stressed group. Based on a study that suggested an increase in physiological indicators such as BP and HR are related to stress [54], the indirect quantification of stress based on behaviours in this study can be interpreted as significant.

Notwithstanding the unexpected result, the present study may suggest a correlation between salivary AVP and acute stress. In the more stressed group, salivary AVP was found to be negatively correlated with BP. Since BP is an indirect measurement of stress, salivary AVP concentration may be negatively correlated with stress. There are few studies in veterinary medicine regarding salivary AVP. Since the results of the current study go against previously known knowledge, researchers should conduct further studies to reveal the nature of salivary AVP, including the relationship between its free and bound forms. After further research, the possibility of salivary AVP as a useful stress biomarker in dogs can be assessed more precisely.

This study has some limitations which should be considered. Firstly, the sample size was small. Despite the given adaptation time, some dogs were still not relaxed after encountering the researcher at home. This could affect pre-stress data. Noise level as a stressor in the present study was chosen considering previously published articles [55, 56]. In one article, 75–78 dB of vacuum noise was enough to decrease salivary sIgA in beagles [56]. However, considering that the present study’s population consisted of privately owned dogs rather than laboratory dogs, it is possible that some of them may have been accustomed to vacuum noise and were not stressed from it but rather from other circumstances. Comparing with a control group without vacuum noise but otherwise identical conditions would make the analysis more obvious, and future studies should take these factors into account. Although the inter-rater reliability for video analysis was excellent, intra-rater reliability was not calculated. The coefficients of variation for ELISA could not be obtained because of limited sample volumes.

## Conclusions

In the current study, salivary AVP decreased after noise- and environmental challenge-stimulated stress. The difference between pre- and post-stress salivary AVP concentration correlated with the difference in BP. In conclusion, our findings suggest that salivary AVP potentially negatively relates to acute stress and necessitates further study as a stress biomarker in dogs.

## Methods

### Subjects

Twenty-eight privately owned, clinically healthy adult dogs were used for this study. All dogs were indoor-living. Dogs included various breeds of both sexes (Table 6). The mean age of the study population was 3.78 ± 2.44 years (ranging from 10 months to 10 years), and the mean body weight was 6.65 ± 4.53 kg (ranging from 2.4–21.4 kg). An online survey was conducted before enrolment to evaluate each dog’s medical history, recent health state, and usual response to stressors (kenneling, vacuum noise, and driving). Their general health was evaluated through routine physical examination, complete blood count, serum chemistry, and urinalysis to exclude any clinically ill dogs. Two dogs showing aggression toward the researcher and four dogs with gingivitis were also excluded. None of the female dogs were pregnant, nursing, or in their oestrus cycle. Consent was obtained from every dog owner for participation in this study. This study was authorised by the Institutional Animal Care and Use Committee of Chungnam National University (approval number: 201906A-CNU-095).

**Table 6:** Characteristics of dog participants

Breed	Number of dogs	Sex of dogs (F/FS/M/MC)
Maltese	8	1/6/0/1
Mixed breed	6	0/4/0/2
Shih Tzu	2	0/2/0/0
Poodle	2	0/0/0/2
Pomeranian	2	0/1/0/1
Yorkshire terrier	1	0/1/0/0
French Bulldog	1	0/1/0/0
Beagle	1	0/1/0/0
Dachshund	1	0/0/0/1
Spitz	1	0/1/0/0
Cocker Spaniel	1	0/0/0/1
Border Collie	1	0/0/0/1
Chihuahua	1	0/0/0/1
**Total**	**28**	**1/17/0/10**

(*F* female, *SF* spayed female, *M* male, *MC* male castrated)

### Study design

This study compared pre- and post-stress vital parameters (BT, HR, RR, and non-invasive systolic BP), salivary AVP, and serum cortisol levels. Defined stress behaviours were analysed by video recording. Since individual stress thresholds may differ and this could affect analysis, physiological and behavioural parameters were used as indirect criteria for each dog’s stress level.

The clinician visited a place where the dog would feel most comfortable (e.g. home) to collect pre-stress samples. After the clinician met the dog, 5–10 min of adaptation time was given to minimise the effect of excitement from a stranger. Sampling was conducted in the less invasive order of RR count, BP measure, HR count, saliva collection, blood collection, BT measure. BP was measured with either the Doppler or oscillometry method, but only one method was employed for each dog. Oscillometry is more inaccurate when the dog is not motionless or has a weak or irregular pulse than the Doppler method. Both Doppler and oscillometry methods are validated for BP determination in conscious dogs when BP is obtained as an average of 5 consecutive measurements by a trained person [57, 58] Five consecutive measurements of BP were conducted by a trained clinician, and the average value was used in the current study.

The dog was moved to the Chungnam National University Veterinary Medical Teaching Hospital by car after the pre-stress sample collection. When the owner’s car was unavailable, the researcher’s was used. Therefore, not all dogs were able to use a familiar car, but in all cases the owner accompanied the dog. The transportation time did not exceed 30 min. The dog was secluded from the owner in a stainless kennel (91 × 56 × 67 cm) in a quiet room. After 10 min of adaptation time, the dog was exposed to vacuum (V-C412T, LG Electronics; Seoul, South Korea) noise for 30 min with video recording (SM-N970, Samsung; Suwon, South Korea) to assess behavioural changes. The vacuum was placed 50 cm away from the kennel. Noise volume was measured at the front of the kennel by a sound level meter (GM1352, Benetech; Shenzhen, China) and adjusted to stay between 85 ~ 95 dB. A clinician stayed in the room during the stress session without social interaction with the dog. Post-stress samples were collected in the same order as pre-stress sampling. All procedures were conducted in the same timeframe (12:00–18:00) to minimise effects of diurnal fluctuation.

A routine physical examination, including dehydration status, auscultation, abdominal palpation, superficial lymph node palpation, and oral cavity inspection, was performed after the stress session finished.

### Stress behaviour analysis

The following actions were defined and analysed as stress behaviours with recorded videos (Table 7) [20, 59]. Shivering and panting, for which frequency was difficult to count, were calculated as a ratio of the duration of the action over 30 min. Oral behaviour, yawning, paw lifting, and vocalisations (bark, howl, and whine) were recorded as frequencies.

**Table 7:** Ethogram of behaviours recorded during 30 min of stress session

Behaviour	Description	Measurement Method
Shivering	Move, shake the body with energy	Duration (% of total record time)
Panting	An increased frequency of inhalation and exhalation often in combination with the opening of the mouth	Duration (% of total record time)
Oral behaviour	Include tongue out: the tip of tongue is briefly extended; snout licking: part of the tongue is shown and moved along the upper lip and/or the mouth; swallowing; smacking	Frequency
Yawning	Slow and deep inhalation through forced and involuntary mouth, jaws and glottis opening with tongue eversion	Frequency
Paw lifting	A forepaw is lifted to a position of approximately 45°	Frequency
Vocalization	Whining: high pitched vocalizations with raised frequency; barking; growling: low frequency vocalizations	Frequency

Videos were analysed by one researcher. To measure observational accuracy, a second researcher analysed five videos using the same ethogram, and inter-rater reliability was calculated.

Since frequency-counting behaviours were displayed in vastly different numerical ranges (i.e. vocalisation; 0 to 1516, yawning; 0 to 15), a simple summing up of the numbers could not reflect dogs’ stress levels adequately. Thus, each behaviour was graded on a scale of 1 to 10 points (1 to 5 for yawning, because of narrow number range), where each behaviour’s average number was in the 5-point range (3 for yawning). Outliers over 10 points (5 for yawning) were given 11 points (6 for yawning). In case of behaviours requiring a duration ratio, every 10% increase was given 1 point. The converted points of each behaviour were summed to constitute a total stress score. Based on the median value of the total score, dogs were divided into two groups: a more stressed group and a less stressed group.

### Saliva sampling and sample storage

SalivaBIO Children’s Swabs (Salimetrics; State College, PA) were used to collect saliva. The clinician held the end of the absorbent stick and made the dog chew or hold it in its cheek pouch for 1–3 min. Owners were instructed not to provide food to their dogs 2 h prior to saliva collection to minimise the effect of food contamination. All saliva and serum samples were centrifuged (saliva: 3000 rpm, 20 min, 4 °C; serum: 6000 rpm, 15 min, room temperature) within 30 min and frozen at − 80 °C immediately after separation until analysis.

### Salivary AVP and serum cortisol quantification

Salivary AVP concentration was measured using a commercially available enzyme-linked immunosorbent assay (ELISA) kit from Enzo Life Science (ADI-900-153A; Farmingdale, NY) which has been previously validated [29]. This assay uses a goat-anti-rabbit IgG antibody-coated microtiter plate with AVP linked to horseradish peroxidase. The cross-reactivities of AVP antibody with related compounds were provided by the manufacturer. The limit of detection of the assay was 2.84 pg/mL. Every sample was assayed in duplicate and not extracted. All procedures followed the product manual.

Serum samples were referred to a laboratory (POBANILAB, Gyeong-gi, South Korea) for serum cortisol analysis. The serum cortisol concentration was measured by ELISA using a VIDAS multiparametric immunoassay system (bioMérieux, Marcy-l’Étoile, France).

### Statistical methods

SPSS Statistics 24 (IBM; Armonk, NY) was used for statistical analysis. A Shapiro-Wilk test (*p* < 0.05) was applied to investigate the normality of pre- and post-stress vital signs (BT, HR, RR, and BP), salivary AVP, and serum cortisol. Paired T-tests or Wilcoxon signed-rank tests were applied for comparing pre- and post-stress parameters. Spearman’s rank correlation or Pearson’s correlation was employed to investigate correlations between parameters.

Considering the individual stress level disparity, statistical analysis was conducted separately in the total study population, the less stressed group, and the more stressed group, respectively.

## Supplementary information

**Additional file 1.**

## Data Availability

Raw data for tables of the current study are available from the corresponding author on reasonable request. Behavioral analysis data processing and prior online survey questionnaire is available as supplementary information file.

## Abbreviations

AVP: Arginine vasopressin
ANP: Atrial natriuretic peptide
BP: Blood pressure
BT: Body temperature
CRF: Corticotropin-releasing factor
CSF: Cerebrospinal fluid
CgA: Chromogranin A
HPA: Hypothalamus-pituitary-adrenal
HR: Heart rate
RR: Respiratory rate
sAA: Salivary alpha amylase
SAM: Sympathetic-adreno-medullar
sIgA: Secretory immunoglobulin A
