# Supplementary material for: Evaluation of salivary vasopressin as an acute stress biomarker in healthy dogs with stress due to noise and environmental challenges
Source: BMC Vet Res. 2020 Sep 11;16:331. doi: 10.1186/s12917-020-02555-5 (PMC7488768; doi:10.1186/s12917-020-02555-5)
Supplement: Supplementary file 1 — Additional file 1. [file 12917_2020_2555_MOESM1_ESM.docx]

1. Behavioral analysis raw data processing procedure

| **No.** | **Recording time** | **Vocalization** | **Mouth licking** | **Yawning** | **Paw lifting** | **Panting** | | **Shivering** | |
| --- | --- | --- | --- | --- | --- | --- | --- | --- | --- |
|  |  |  |  |  |  | **Time** | **%** | **Time** | **%** |
| **1** | 30:25:00 | 34 | 131 | 7 | 1 | 19:52 | 65.31507 | 26:27:00 | 86.9589 |
| **2** | 29:52:00 | 0 | 99 | 15 | 9 | 6:03 | 20.2567 | 17:13 | 57.64509 |
| **3** | 30:45:00 | 591 | 22 | 0 | 18 | 0 | 0 | 0 | 0 |
| **4** | 30:00:00 | 973 | 16 | 0 | 1 | 30:00:00 | 100 | 0 | 0 |
| **5** | 29:25:00 | 206 | 147 | 7 | 2 | 2:27 | 8.328612 | 5:52 | 19.94334 |
| **6** | 30:09:00 | 0 | 7 | 2 | 0 | 0 | 0 | 0 | 0 |
| **7** | 30:11:00 | 984 | 19 | 3 | 63 | 25:52:00 | 85.69851 | 0 | 0 |
| **8** | 31:50:00 | 0 | 46 | 4 | 0 | 0:32 | 1.675393 | 0 | 0 |
| **9** | 34:40:00 | 0 | 46 | 0 | 0 | 0 | 0 | 0 | 0 |
| **10** | 30:47:00 | 0 | 21 | 0 | 0 | 0 | 0 | 0 | 0 |
| **11** | 29:37:00 | 11 | 100 | 0 | 0 | 0 | 0 | 0 | 0 |
| **12** | 32:36:00 | 16 | 13 | 1 | 5 | 0 | 0 | 0 | 0 |
| **13** | 32:36:00 | 0 | 2 | 1 | 0 | 0 | 0 | 0 | 0 |
| **14** | 30:04:00 | 0 | 105 | 13 | 4 | 0 | 0 | 25:01:00 | 83.20399 |
| **15** | 30:04:00 | 0 | 18 | 0 | 1 | 0 | 0 | 29:33:00 | 98.2816 |
| **16** | 31:22:00 | 158 | 23 | 2 | 2 | 0 | 0 | 3:36 | 11.47715 |
| **17** | 30:16:00 | 0 | 28 | 0 | 0 | 0 | 0 | 2:34 | 8.480176 |
| **18** | 31:22:00 | 7 | 114 | 0 | 0 | 0 | 0 | 31:06:00 | 99.14984 |
| **19** | 30:16:00 | 133 | 392 | 1 | 12 | 25:48:00 | 85.24229 | 0 | 0 |
| **20** | 30:19:00 | 265 | 130 | 0 | 15 | 3:34 | 11.76471 | 9:39 | 31.83068 |
| **21** | 30:08:00 | 533 | 168 | 0 | 0 | 30:06:00 | 99.88938 | 23:10 | 76.88053 |
| **22** | 30:25:00 | 0 | 10 | 2 | 2 | 0 | 0 | 0 | 0 |
| **23** | 30:13:00 | 636 | 55 | 0 | 62 | 25:45:00 | 85.21787 | 0 | 0 |
| **24** | 31:36:00 | 1516 | 41 | 0 | 22 | 27:33:00 | 87.18354 | 0 | 0 |
| **25** | 31:47:00 | 0 | 33 | 0 | 0 | 0 | 0 | 29:38:00 | 93.23545 |
| **26** | 31:05:00 | 0 | 95 | 5 | 67 | 22:56 | 73.78016 | 0 | 0 |
| **27** | 30:02:00 | 0 | 411 | 3 | 0 | 21:50 | 72.697 | 29:55:00 | 99.61154 |
| **28** | 30:02:00 | 128 | 100 | 6 | 0 | 2:55 | 9.711432 | 2:50 | 9.433962 |
|  |  | Frequency measure | | | | Duration measure | | | |

- Raw data of video analysis
- Scoring of each behavior

| **Vocali** | **Mouth licking** | **Yawning** | **Paw lifting** |
| --- | --- | --- | --- |
|  |  |  |  |
| 0 | 2 | 0 | 0 |
| 0 | 7 | 0 | 0 |
| 0 | 10 | 0 | 0 |
| 0 | 13 | 0 | 0 |
| 0 | 16 | 0 | 0 |
| 0 | 18 | 0 | 0 |
| 0 | 19 | 0 | 0 |
| 0 | 21 | 0 | 0 |
| 0 | 22 | 0 | 0 |
| 0 | 23 | 0 | 0 |
| 0 | 28 | 0 | 0 |
| 0 | 33 | 0 | 0 |
| 0 | 41 | 0 | 1 |
| 7 | 46 | 1 | 1 |
| 11 | 46 | 1 | 1 |
| 16 | 55 | 1 | 2 |
| 34 | 95 | 2 | 2 |
| 128 | 99 | 2 | 2 |
| 133 | 100 | 2 | 4 |
| 158 | 100 | 3 | 5 |
| 206 | 105 | 3 | 9 |
| 265 | 114 | 4 | 12 |
| 533 | 130 | 5 | 15 |
| 591 | 131 | 6 | 18 |
| 636 | 147 | 7 | 22 |
| 973 | 168 | 7 | 62 |
| 984 | 392 | 13 | 63 |
| 1516 | 411 | 15 | 67 |
| 221 | 85 | 2.5 | 10.2 |

The green boxes are the average of each behaviours, and the yellow boxes are closest raw values to each average.

We set yellow box numbers as 5 points out of 10 (3 out of 5 for yawning, because numbers were too small to divide to 10 grades). Based on this, we set the range of each grade.

Also, if the number of the behaviours was over 10 points (5 points for yawning), those were classified as outliers, and given 11 points (6 for yawning).

For duration record behaviours, a 10% increase was equal to 1 point.

The box below shows the grading criteria for behaviours as a result.


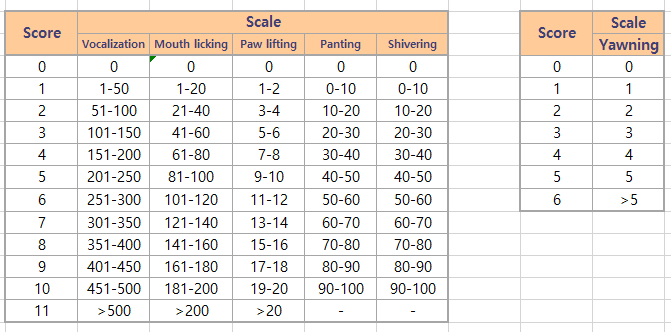


- The final data of behavior analysis after raw data processing.

| No. | Vocalization | Mouth licking | Yawning | Paw lifting | Panting | Shivering | **Score SUM** |
| --- | --- | --- | --- | --- | --- | --- | --- |
| **1** | 1 | 7 | 6 | 1 | 7 | 9 | **31** |
| **2** | 0 | 5 | 6 | 5 | 3 | 6 | **25** |
| **3** | 11 | 2 | 0 | 9 | 0 | 0 | **22** |
| **4** | 11 | 1 | 0 | 1 | 10 | 0 | **23** |
| **5** | 5 | 8 | 6 | 1 | 1 | 2 | **23** |
| **6** | 0 | 1 | 2 | 0 | 0 | 0 | **3** |
| **7** | 11 | 1 | 3 | 11 | 9 | 0 | **35** |
| **8** | 0 | 3 | 4 | 0 | 1 | 0 | **8** |
| **9** | 0 | 3 | 0 | 0 | 0 | 0 | **3** |
| **10** | 0 | 2 | 0 | 0 | 0 | 0 | **2** |
| **11** | 1 | 5 | 0 | 0 | 0 | 0 | **6** |
| **12** | 1 | 1 | 1 | 3 | 0 | 0 | **6** |
| **13** | 0 | 1 | 1 | 0 | 0 | 0 | **2** |
| **14** | 0 | 6 | 6 | 2 | 0 | 9 | **23** |
| **15** | 0 | 1 | 0 | 1 | 0 | 10 | **12** |
| **16** | 4 | 2 | 2 | 1 | 0 | 2 | **11** |
| **17** | 0 | 2 | 0 | 0 | 0 | 1 | **3** |
| **18** | 1 | 6 | 0 | 0 | 0 | 10 | **17** |
| **19** | 3 | 11 | 1 | 6 | 9 | 0 | **30** |
| **20** | 6 | 7 | 0 | 8 | 2 | 4 | **27** |
| **21** | 11 | 9 | 0 | 0 | 10 | 8 | **38** |
| **22** | 0 | 1 | 2 | 1 | 0 | 0 | **4** |
| **23** | 11 | 3 | 0 | 11 | 9 | 0 | **34** |
| **24** | 11 | 3 | 0 | 11 | 9 | 0 | **34** |
| **25** | 0 | 2 | 0 | 0 | 0 | 10 | **12** |
| **26** | 0 | 5 | 5 | 11 | 8 | 0 | **29** |
| **27** | 0 | 11 | 3 | 0 | 8 | 10 | **32** |
| **28** | 3 | 5 | 6 | 0 | 1 | 1 | **16** |

The mean value of the summed score was 18.25. Therefore, we defined dogs with over 18.25 points as more stressed and the rest as les stressed.

| Stress level | Score SUM | Number of dogs |
| --- | --- | --- |
| more | Over 18.25 | 14 |
| less | Less than 18.25 | 14 |

1. Online survey

- Please write your name and phone number.
- Please write down the address (home, office, etc. where your dog feels most comfortable) that researchers can visit.
- General Questions

1. What is your dog’s name?
2. How old is your dog? (e.g. ~ y ~m)
3. Let us know your dog’s sex status.
   1. Female
   2. Male
   3. Female spayed
   4. Male castrated
4. If you know, let us know breed of your dog
5. Are there any diseases you are currently managing or medications you are administering to your dog?
   1. Yes (go to question number 5-1)
   2. No (go to question number 6)

5-1. What is the disease or medicine?

1. Has the dog ever got surgery or been hospitalized? (Excepting neutralization surgery)
   1. Yes (go to question number 6-1)
   2. No (go to question number 7)

6-1. What was the disease or surgery?

1. Has dog’s appetite changed in the last month?
   1. Not changed (good)
   2. More than usual
   3. Less than usual
2. Has dog’s vitality changed in the last month?
   1. Not changed (good)
   2. More than usual
   3. Less than usual
3. Has there been a change in the amount of drink and urination in the last month?
   1. Not changed
   2. More than usual
   3. Less than usual
4. What's the frequency and location of dog’s usual walk?

- Questions about kenneling

1. Has your dog ever used Kennel?
   1. Yes (go to question number 1-1)
   2. No (go to question number 2)
   3. Has the dog been got house training using Kennel at home?
      - - 1. Yes (use as dog’s own room at home)
          2. No (use only on the move)
   4. How often do you use Kennel? (Included in the home, on the move/on a week or month basis)
   5. What is the average usage time of Kennel?
2. Less than 30 minutes
3. More than 30 minutes
   1. How much stress do you feel when the dog is using Kennel?
4. The dog is so stressed out that it can't stand being in Kennel.
5. The dog seems to be under stress, but it manages to endure it well
6. The dog can wait very comfortably inside Kennel.
7. Has your dog ever used a carrier other than Kennel?
   1. Yes (go to question number 2-1)
   2. No (go to question number 3)

2-1. What type of carrier do you usually use?

2-2. Has the dog been got house training using the carrier at home?

2-3. How much stress does your dog feel when using Kennel?

1. The dog is so stressed out that it can't stand being in Kennel.
2. The dog seems to be under stress, but it manages to endure it well
3. The dog can wait very comfortably inside Kennel.

- Questions about car boarding and vacuum noise

1. Has your dog ever traveled in a car?
   1. Yes (go to question number 1-1)
   2. No (go to question number 2
   3. How many times have your dog been in a car? Or how often is it?
   4. Do you use Kennel or carrier when the dog is using a car?
      - 1. Yes
        2. No
   5. How much stress does your dog feel when riding a car?
      - - 1. The dog is so stressed out that it can't stand being in a car.
          2. The dog seems to be under stress, but it manages to endure it well
          3. The dog can wait very comfortably inside a car.
2. How much stress does your dog feel when you use a vacuum cleaner at home?
   1. The dog is very stressed and afraid of the vacuum
   2. The dog seems to be under stress, but it manages to endure it well
   3. The dog doesn’t care at all about the noise of the vacuum.
